# Data Safe Havens in health research and healthcare

**DOI:** 10.1093/bioinformatics/btv279

**Published:** 2015-06-25

**Authors:** Paul R. Burton, Madeleine J. Murtagh, Andy Boyd, James B. Williams, Edward S. Dove, Susan E. Wallace, Anne-Marie Tassé, Julian Little, Rex L. Chisholm, Amadou Gaye, Kristian Hveem, Anthony J. Brookes, Pat Goodwin, Jon Fistein, Martin Bobrow, Bartha M. Knoppers

**Affiliations:** ^1^Data to Knowledge (D2K) Research Group, University of Bristol, Oakfield House, Oakfield Grove, Clifton, Bristol BS8 2BN, UK,; ^2^Public Population Project in Genomics and Society (P^3^G), Montreal, QC H3A 0G1, Canada,; ^3^Department of Computer Science, University of Toronto, Sandford Fleming Building, Toronto, ON M5S 3G4, Canada,; ^4^JK Mason Institute for Medicine, Life Sciences and the Law, School of Law, University of Edinburgh, Old College, South Bridge, Edinburgh EH8 9YL, UK,; ^5^Department of Health Sciences, University of Leicester, Adrian Building, University Road, Leicester LE1 7RH, UK,; ^6^School of Epidemiology, Public Health and Preventive Medicine, Faculty of Medicine, University of Ottawa, Ottawa, ON K1H 8M5, Canada,; ^7^Center for Genetic Medicine and Surgery, Northwestern University, Rubloff Building, 750 N Lake Shore, Chicago, IL 60611, USA,; ^8^Department of Public Health and General Practice, Norwegian University of Science and Technology, Postboks 8905, 7401 Trondheim, Norway,; ^9^C3 Collaborating for Health, 7-14 Great Dover Street, London SE1 4YR,; ^10^MRC Medical Bioinformatics Centre, Leeds Institute of Health Sciences, University of Leeds, University of Leeds, Charles Thackrah Building,101 Clarendon Road, Leeds LS2 9LJ,; ^11^University of Cambridge, Wolfson College, Cambridge CB3 9BB, UK and; ^12^Centre of Genomics and Policy, McGill University, Montreal, QC H3A 0G1, Canada

## Abstract

**Motivation:** The data that put the ‘evidence’ into ‘evidence-based medicine’ are central to developments in public health, primary and hospital care. A fundamental challenge is to site such data in repositories that can easily be accessed under appropriate technical and governance controls which are effectively audited and are viewed as trustworthy by diverse stakeholders. This demands socio-technical solutions that may easily become enmeshed in protracted debate and controversy as they encounter the norms, values, expectations and concerns of diverse stakeholders. In this context, the development of what are called ‘Data Safe Havens’ has been crucial. Unfortunately, the origins and evolution of the term have led to a range of different definitions being assumed by different groups. There is, however, an intuitively meaningful interpretation that is often assumed by those who have not previously encountered the term: a repository in which useful but potentially sensitive data may be kept securely under governance and informatics systems that are fit-for-purpose and appropriately tailored to the nature of the data being maintained, and may be accessed and utilized by legitimate users undertaking work and research contributing to biomedicine, health and/or to ongoing development of healthcare systems.

**Results:** This review explores a fundamental question: ‘what are the specific criteria that ought reasonably to be met by a data repository if it is to be seen as consistent with this interpretation and viewed as worthy of being accorded the status of ‘Data Safe Haven’ by key stakeholders’? We propose 12 such criteria.

**Contact:** paul.burton@bristol.ac.uk

## 1 Introduction

### 1.1 Data in society

We live in a data-rich and increasingly information-driven world, and society is rapidly responding to the opportunities and challenges this presents (Davies *et al.*, 2002; Shaw, 2014). This is as true in biomedicine as in any other domain of human endeavour (Lohr, 2012; OECD Expert Group for International Collaboration on Microdata Access, 2014). Public expectations of health services have never been higher, with greater emphasis on ensuring accountability, effectiveness, efficiency, quality and safety of health services (Wyke, 2009). But, these expectations can never be met in full because demands on health services typically exceed the resources that societies make available (Calabresi and Bobbitt, 1978). The management of health and disease, policy and decision making and the development of healthcare systems in this resource-limited environment demand underpinning by evidence-based investigation and evaluation that is as rigorous as is currently possible. The data that put the ‘evidence’ in ‘evidence-based medicine’ are therefore central to developments in public health, primary and hospital care, for both generic and personalized/stratified medicine (Academy of Medical Sciences, 2013; Shaw, 2014).

Relevant data may be collected as part of usual healthcare, from other routine administrative sources or primarily for research purposes projects (Academy of Medical Sciences, 2014). These data may be used to guide decisions and management in healthcare or to drive research to inform future decisions and planning. The generation and use of these data are embedded in complex, and ever-changing, social settings, structures and networks: from individual researchers or research groups to international research consortia and from individual healthcare practitioners or institutions to whole-of-country health systems. The management and utilization of healthcare and health research data comprise socio-technical solutions (Geels, 2005) that may easily become enmeshed in controversy as they encounter the norms, values, expectations and fears of diverse stakeholders. While some commentators (e.g. data generators, many healthcare professionals, research users, funders, industry, and many in government) may argue the benefits of appropriately analysed and interpreted data, others may express some degree of ambivalence or outright concern regarding the collection and use of personal data to inform evidence-based healthcare. Thus, worries about risks to individual privacy and confidentiality are played out against potential population benefits. This tension, often described in terms of the balance of individual rights and public good, is well evidenced in recent controversies in the UK following the attempted introduction by the NHS (National Health Service) of a nationwide mechanism, branded as ‘care.data’, for making routinely collected individual-level health data available for research (Taylor, 2014). The care.data scheme was postponed following a media furore that characterized the proposal as, at one extreme, the ‘big brother data plan’ (Adams, 2013) and, at the other, ‘a basic human right’ (Kelsey, 2013). Notwithstanding the hyperbole of media constructions, this so-called ‘crisis’ is consistent with the rise of an increasingly data-savvy general public that is wary of expert claims to appropriately care for their personal information.

The ‘individual rights/public good’ dichotomy is based on a Western-centric view of *individual autonomy* that is pervasive but not ubiquitous, and not necessarily useful for purposes of appropriately managing human health and research data. Autonomy is understood as the right (and capacity) of an individual to *decide for themselves*, but that right, the *right to choose*, is conditional on broader consideration of the impact of those decisions on others (Beauchamp and Childress, 1979). In most Western conceptualizations it is the former condition that is emphasized in combination with the principle of *non-maleficence* (Beauchamp and Childress, 1979) which places great moment on ensuring that no individual is unknowingly placed at risk of harm from research or healthcare—regardless how low that risk actually may be nor how minor the potential harm. A narrow view of autonomy, however, ultimately constrains patient and public decisions in healthcare (Murtagh and Hepworth, 2003). Furthermore, if it is accorded undue weight by ethical committees it can sometimes inhibit valuable scientific work. This may happen, for example, if ethics committees insist on obtaining formal consent when it is difficult to obtain in practice or counterproductive from a scientific perspective (Hansson, 2010), and/or in any setting where the public and patients understand the reasons for, and are supportive of, ‘research without consent’ because it brings direct public benefit (Lecouturier *et al.*, 2008). Under such circumstances, adoption of a broader definition of autonomy—which includes the *right to contribute to society* if one so chooses—might significantly facilitate important health related, particularly data-driven, research (Hansson, 2010). Going further - beyond the consideration of *rights*—a handful of commentators have argued that individuals who benefit from the provision of sophisticated healthcare have a *responsibility* to allow their personal data to be used to help the development of that system (Doll and Peto, 2001).

Wherever you stand in debates about freedom of the individual, the fact remains that, as researchers and policy makers in health, we have an ethical and legal duty to ensure that data are managed and used as effectively as possible. But we should not be disingenuous in this stance; along with the ethico-social imperatives there are clear pragmatic benefits to ‘taking responsibility’, not least in facilitating our own research practice. Being responsive to the evolving societal context—engaging the range of governance and other mechanisms by which to do this—is fundamental to maintaining the relationship of *trust* that is required to ensure public and research participants have sufficient confidence to provide the data upon which research and healthcare development depend. Appropriate and proportionate governance and stewardship of the processes of data for health research require a systemic approach which includes but goes beyond narrowly defined formal mechanisms of responsibility (Owen *et al.*, 2013). Such an approach, focusing on the establishment and maintenance of *trustworthiness*, would necessarily be aligned with broad societal norms and values, embody the collective responsibility of science *and* society, and would be responsive and dynamic.

### 1.2 What’s in a name? The creation of ‘Data Safe Havens’

Given the need to ensure that health-related data are used efficiently, effectively and in a manner that is socially aware, a fundamental challenge is to site them in repositories that can easily be accessed by those with the need and permission to do so. However, they must be under appropriate technical and governance controls to ensure security and these must be properly evaluated and audited. In this context, the development of what are called ‘Data Safe Havens’ has become increasingly popular (Nuffield Council on Bioethics, 2015). The first formal use of this title in relation to the management of health data appears to have been by the British National Health Service (NHS) in the early 1990s: ‘safe haven’ being used to cover both a defined physical location and an administrative set of policies and procedures relating to the secure handling of confidential patient information (Directorate of Information Services MEL(1992)42, 1992). Since then the term has evolved in at least three different directions (Administrative Data Taskforce, 2012; Caldicott, 2013; NHS Research Capability Programme, 2008): (i) *a specific term* to reflect a changing series of different data management constructs primarily related to the NHS and with a particular focus on record linkage—e.g. ‘a protected space under the control of an independent clinician’ (Anderson, 1996); secure environments, limited to small numbers of users, in which data are managed, quality is assessed and linkage between records can take place (NHS Research Capability Programme, 2008); and a means ‘to ensure the safety and secure handling of confidential patient identifiable information’ (NHS Connecting for Health, 2009); (ii) *a more generic term*, subsuming for example the Data Safe Haven at University College London, that implies ‘a designated physical or electronic area that provides the most appropriate level of security for the use of the most sensitive and confidential information’ (Care Record Development Board, 2007), or ‘an environment for population-based research and statistical analysis in which the risk of identifying individuals is minimized’ (Thomas *et al.*, 2008) or a ‘physical environment where access to disclosive data can be controlled’ (Administrative Data Taskforce, 2012); and (iii) *specialist secure settings* in which data can be analysed—either locally, or remotely via secure privacy protecting mechanisms, but cannot physically be removed from that setting (Lyons, 2009 #5082; Administrative Data Taskforce, 2012 #5799; Academy of Medical Sciences, 2014 #5787; Jones, 2014 #5821) NHS-Scotland National Safe Haven (http://www.adls.ac.uk/nhs-scotland/nhs-scotland-national-safe-haven/). In this third category, it should be noted that the OECD microdata report uses the terms ‘data enclave’ or ‘safe centre’ for the specific case of ‘a facility equipped with computers not linked to the internet or an external network and from which no information can be downloaded via USB ports, CD-DVD or other drives’ (OECD Expert Group for International Collaboration on Microdata Access, 2014).

Unfortunately, for the reasons outlined above there is now serious uncertainty about appropriate usage of the term ‘Data Safe Haven’. Furthermore, although the various meanings have evolved primarily within the UK, it is increasingly being used internationally (Global Alliance for Genomics and Health, 2014). This adds an additional potential for confusion with the ‘Safe Harbour Principles’ that relate to cross-border data transfer rules under EU Directive 95/46/EC on the protection of personal data. Recognizing the potential for serious confusion, the UK Academy of Medical Sciences recently ran an international workshop on Data Safe Havens (Academy of Medical Sciences, 2014). It was concluded that, from a generic perspective, they are provided to ‘enable… [data] access and linkage’ whilst ‘upholding the duty of confidentiality and protecting the data subject’s right to privacy’ (Academy of Medical Sciences, 2014). But at the same time, it was noted that ‘agreeing on a single definition of ‘data safe haven’ will be difficult as there is a wide variety of systems in operation’. Moreover, there is, for example, ‘no consistency on whether the safe haven: holds identified or de-identified data; provides access to data on site or remotely; processes data and sends them externally; and provides training and support for data users’ (Academy of Medical Sciences, 2014). This lack of consensus about the detailed implications of a term that is of such potential value may in part explain why the phrase ‘safe haven’ is completely absent from the comprehensive recent OECD review of international access to microdata (OECD Expert Group for International Collaboration on Microdata Access, 2014). The Global Alliance for Genomics and Health has therefore adopted *Data Safe Havens* as a specific focus for its Regulatory and Ethics Working Group and this current paper reflects some of the key thinking arising from that decision, particularly through its foundational *Framework for Responsible Sharing of Genomic and Health-Related Data* (Global Alliance for Genomics and Health, 2014).

From a pragmatic perspective, the definitional ambiguity we have highlighted may be viewed as less than ideal but not fatal. Yet that would be to underestimate the impact that use—or non-use—of specific language can have on society’s perspective of challenging concepts and agencies. In this regard, few would argue against society developing *safe havens for data* in a sense that would be meaningful and valid both to professionals and to the general public: i.e. repositories in which useful but potentially sensitive data could be kept securely allowing them to be used by legitimate professionals undertaking work and research contributing to biomedicine, health and to ongoing development of the healthcare system. From this perspective, which is based on an intuitive interpretation, the term ‘Data Safe Haven’ is therefore an appellation that could be used to very good purpose but must be wielded with caution. If entities called ‘Data Safe Havens’ were to turn out to have characteristics that worried a substantial number of individuals or society as a whole or if they were to fail to come up to the standards implied by their name, the very term could start to take on negative connotations. The ease with which this may occur is clearly demonstrated by the care.data example, given earlier.

Unfortunately, the current definitional ambiguity, coupled with the fact that some users of the term *do* believe it to have a single specific meaning, has meant that two professionals discussing *Data Safe Havens* can completely misunderstand one another and yet to have no reason to suspect that a misunderstanding has arisen. Arguably, therefore, the undeniable importance of the underlying concept and the potential value of the term itself, imply that this uncertainty demands urgent resolution. This paper attempts to do this. However, we approach the problem from a rather different standpoint than other recent attempts to clarify the definition (Academy of Medical Sciences, 2014). To be specific, we start from the position that there is *no universally accepted definition of the term*—even if it may have been clearly defined at the outset (Directorate of Information Services MEL(1992)42, 1992) and may have a particular legal interpretation in certain jurisdictions (Caldicott, 2013). In consequence, there is little point in trying to identify a ‘correct’ definition which may be contrasted with other ‘incorrect’ definitions. Instead, this paper addresses the more basic, and in our view more important, question: *what are the specific challenges that ought reasonably to be met by a data repository in order that the researchers managing and using it, the individuals who originally provided their data and other key stakeholders might reasonably be expected to agree that the repository is ‘trustworthy’ in that it is managed and used in a manner that maintains acceptable data integrity and ensures their appropriate security?*

In identifying these challenges we consider whether there is a particular constellation of criteria that might be viewed as defining an entity that could reasonably be called a ‘Data Safe Haven’. However, to begin, we describe the phenomenon that we understand to provide the contextual underpinning for discussion of Data Safe Havens: the data pipeline.

## 2 The data pipeline in contemporary health science

**Fig. 1.**
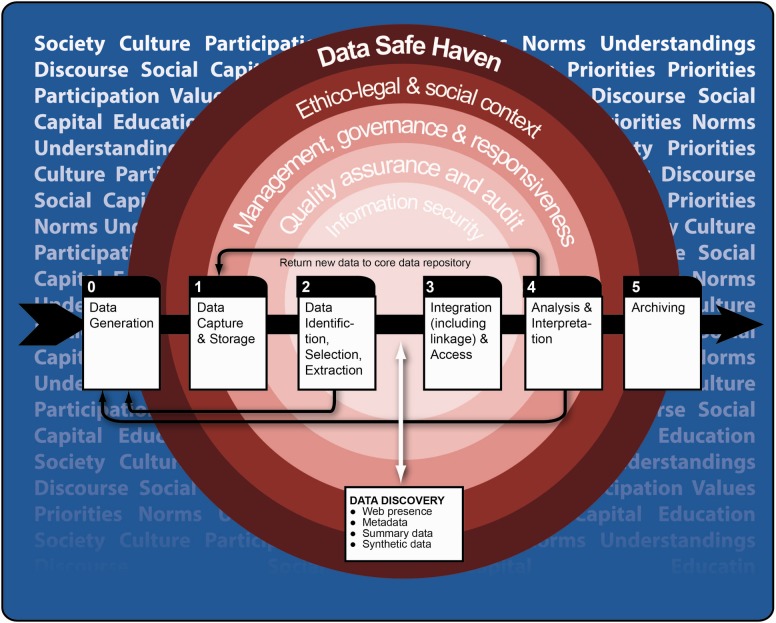
The data pipeline

The term ‘data pipeline’ is used widely; in the computing setting it typically refers to ‘*a chain of data-processing stages…’* (e.g. (Dilger *et al.*, 2013). Here, we use it—with this same basic interpretation—to refer to a simplified conceptual representation of the life-course of individual-level data in a biomedical data repository (a location for the secure storage of biomedical research and/or healthcare-related data) from the moment of collection or generation, through their utilization and conversion into useful knowledge, and ultimately their archiving or destruction. At its simplest, this data pipeline may be viewed as comprising two primary components that are temporally and sometimes spatially distinct: acquisition and exploitation (Fig. 1). *Acquisition* subsumes the processes of data generation, capture, storage and archiving whereby data originating in human and social contexts are amassed in a data repository. *Exploitation* refers to the means by which these data are processed and managed to be readied for access for health service provision, audit and evaluation and/or by research users for analysis and interpretation which can itself generate individual-level data which may be returned to the repository. Analysis and interpretation may span a broad spectrum involving hypothesis- or model-driven methods and/or data mining and hypothesis-free approaches to knowledge discovery.

It is the need to properly manage the flows of data along the data pipeline as well as into and between *acquisition* and *exploitation* that creates the necessity for trustworthy research environments within which to house the requisite systems and processes, and such environments may conveniently be called Data Safe Havens (DSHs). The three words constituting this term hint at the fundamental physical nature of these entities and, at the same time, imply an important dichotomy of primary aims. As a physical entity a Data Safe Haven is a physical repository for data that occupies a defined location in space and across time. As to its purpose, it has two complementary roles: (i) to manage information in a manner that ensures *data* fidelity, *data* quality and *data* utility; and (ii) to keep the data *safe* in the sense that they are used in ways that are consistent with all applicable governance considerations and avoid harm or distress for research participants (data contributors), generators, users or for the repository itself. In this sense a Data Safe Haven may be seen as a collection of structures and processes that ensure data integrity and make them available for research, healthcare or other secondary processes such as population health monitoring and health service planning. But this simple definition needs some elaboration, specifically in relation to the possible mechanisms for access; this might lead us to recognize different classes of Data Safe Haven.

There are three fundamental ways that individual-level data [=*‘*microdata’ (OECD Expert Group for International Collaboration on Microdata Access, 2014)]—or the information contained in such data—might reasonably be accessed. First, the data themselves could be held in a repository and released to potential users, with or without governance controls on that release. At present this class of data repository is the commonest in the field of biomedical and health science (e.g. NHS Health and Social Care Information Centre [http://www.hscic.gov.uk], 1958 Birth Cohort (Power and Elliott, 2006), CARTaGENE [www.cartagene.qc.ca/en/researcher-access], dbGaP [www.ncbi.nlm.nih.gov/gap], European Genome-phenome Archive [https://www.ebi.ac.uk/ega/], ICGC Data Portal [https://dcc.icgc.org/], UK Biobank (Collins and and UK Biobank Steering Committee, 2007), UK Data Archive [www.data-archive.ac.uk]). Second, the data could be held in a repository which users can access to analyse those data, but they cannot see or extract the data, and the analytic routines which are available to them may include formal disclosure controls—e.g. no release of contingency tables if any cell count is between 1 and 4. Approaches to doing this already exist and include systems that vary from major IT infrastructural projects such as the UK Data Service Secure Lab [http://ukdataservice.ac.uk/use-data/secure-lab.aspx]), to open source solutions such as DataSHIELD (Gaye *et al.*, 2014; Wolfson *et al.*, 2010) and ViPAR (Chi, 2013). Approaches under either of these first two classes may require users to physically visit the location where the servers holding the data are based (NHS Scotland National Safe Haven, 2014; {OECD Expert Group for International Collaboration on Microdata Access, 2014 #5792}, or may involve appropriately secured remote access (Gaye *et al.*, 2014; Karr *et al.*, 2007). A third class of repository might directly release data to applicants, but in a modified form that mitigates disclosure risk. As examples, this may include: restriction of data release to study-level summary statistics; lesser degrees of data collapse involving resolution into tables that are non-disclosive (Di Iorio *et al.*, 2013) possibly with formal control of disclosure risk via k-anonymization (Sweeney, 2002); addition of random noise [e.g. noise-based differential privacy mechanisms (Dwork, 2006)] or the generation of synthetic data (simulated data with an equivalent joint distribution to a set of real data; Karr and Reiter, 2014). Each of these *classes* of Data Safe Haven may require different mechanisms to ensure that they are *safe.*

All of these models of sharing biomedical data have *pros* and *cons.* For example, there may be some loss of information content from the original data and *no* approach to data release can ever completely guarantee that disclosure is impossible. But it is our view that provided a repository meets the criteria defining data quality and safety that are appropriate to the particular data it holds, all such approaches should have the potential to be viewed as a Data Safe Haven. It is true that some of these may focus particularly closely on data security while others may focus on streamlining and simplifying data access, but provided they are compliant with the key criteria, and are *fit for purpose* examples from all classes might reasonably be viewed as Data Safe Havens.

## 3 What makes for a Data Safe Haven?

Regardless of its origins and initial meaning, common and intuitive usage of the term suggests that if an entity is to be viewed as a Data Safe Haven, it must first be able to store and release *data* faithfully and effectively. Second, it must be able to do this in a manner that might reasonably be viewed as *safe* and *trustworthy* by all key stakeholders: e.g. research participants (data contributors), data generators, repository staff, data users, research ethics committees, etc.

To formalize the process of determining whether a particular data repository is ‘faithful to its data’ and that all systems and processes may be regarded as safe, it might reasonably be argued that a series of specific but flexible and responsive criteria should be met: data maintenance and access processes must be socially acceptable and appropriate; any use must be based on data that are veritable, meaning that they must be maintained and released in a form that is faithful to their origins, and; to warrant the moniker ‘safe’, it must be secure and must be seen to be secure. In other words, a Data Safe Haven should be trustworthy and its underlying systems and policies should operate in an entirely transparent manner. With further development and evaluation, these same criteria for describing Data Safe Havens may also be considered criteria for assessing the status of a repository. A preliminary set of criteria is presented below (Table 1). However, it is important to recognize that we do not claim these are the optimal or the only criteria to use. Rather, we believe these criteria provide a starting point for defining and identifying trustworthy research environments that might be worthy of the title ‘Data Safe Haven’. They also provide a framework for formally evaluating and accrediting such safe havens.

**Table 1.** Proposed criteria for a Data Safe Haven

**Data maintenance and release should be socially acceptable and appropriate**	
Criterion 1	Consistent with formal ethical and legal requirements
Criterion 2	Responsive to emerging issues
Criterion 3	Discoverable and accessible
Criterion 4	Transparent and accountable
**Data should be veritable**	
Criterion 5	Data and metadata fidelity
Criterion 6	Quality assurance and control
Criterion 7	Curation and archiving
Criterion 8	Reliable availability including backup
Criterion 9	Effective audit
**Data should be safe and secure**	
Criterion 10	Preserve confidentiality, integrity and availability of the repository
Criterion 11	Appropriate secure access to individually identifying data
Criterion 12	Appropriate protection of individually identifying data

### 3.1 Data maintenance and release must be socially acceptable and appropriate

1. *Consistent with formal ethical and legal requirements.* This includes compliance with relevant national and international legislation (e.g. data protection legislation), consents, information documentation and any other ethical, legal or governance controls that applied when the data were originally generated (including from biosamples). It also includes meeting formal governance requirements that may apply to the repository itself or to users of the data, e.g. formal permission from a data access committee, formal data transfer agreements, definitions of a *bona fide* user.
2. *Responsive to emerging issues, e.g. whether to return clinically relevant research results.* Appropriate mechanisms and systems must be in place to respond in a timely, inclusive (i.e. involving all relevant stakeholders) and appropriate manner to emerging socio-technical (e.g. introduction of a new technology or analytic method with potential to impact identifiability) or socio-ethical (e.g. analysis which reveals clinically relevant findings) issues.
3. *Discoverable and accessible.* It must be possible—and ideally straightforward—for potential users to find out what data are held by a repository and how to apply to access them. This should include up-to-date provision of readily accessible metadata.
4. *Transparent and accountable.* All policies and written agreements underpinning a repository’s processes for data management (including any legal contracts under Criterion 1) should be properly documented, and freely available to anybody upon whom they may impact (including data participants). This should include up-to-date provision of repository policies, application forms and any legal controls, e.g. data transfer agreements, or data protection statutes that may apply. In particular, any decisions regarding access to data should be based on formally stated criteria that are fit-for-purpose—neither too restrictive nor too *laissez faire* –readily interpretable and are seen to be fairly applied. In case of dispute regarding an access decision, there should be a transparent and independent appeals process. There should also be clearly specified sanctions that would apply in the case of violation of an agreed principle; sanctions which should be appropriately calibrated to the nature of the violations that have necessitated them. This is a developing area.

### 3.2 Data must be veritable

5. *Data and metadata fidelity.* The data and metadata that may be accessed from a repository should directly reflect the data that were originally lodged in that repository, or should reflect an agreed transformation (or recorded update/correction) of those original data. This should be true regardless when or how those data may later be accessed.
6. *Quality assurance and control.* Appropriate quality assurance systems should be in place to facilitate identification and subsequent correction of data errors.
7. *Curation and archiving.* All data and systems must be effectively maintained to ensure digital continuity and that systems do not become obsolete or the data irretrievable and should include sufficient documentation and metadata to allow users to interpret data in the context in which they were collected.
8. *Effective backup routines.* All data and systems should be appropriately and regularly backed up.
9. *Effective audit.* If a repository is to be relied upon as a safe source of high quality data, it is important not only that it formally meets all of the criteria considered above, but that it is possible for it to *demonstrate* that these criteria are met and continue to be met. Ideally this should be assessed through independent assessment. This demands an effective audit program. Auditing is also one of the main tools for profiling secondary uses of data by authorized users.

### 3.3 Data must be safe and secure

10. *Preserve confidentiality, integrity and availability of the repository.* All systems and resources should have appropriate safeguards to preserve confidentiality, integrity and availability of the repository. These should include physical, administrative, and technical controls such as secure storage facilities, key/password management procedures, firewalls, virus scanners, audit logging and non-repudiation mechanisms. Safeguards (e.g. appropriate encryption) should be designed to protect data in transit, whether inside or outside the trust boundary. Security safeguards and management systems should meet relevant standards (e.g. ISO/IEC 27000-series). A repository should also enforce basic security measures on data users—e.g. by making the award of data conditional on the data being kept on a password protected server—these measures would be explicitly documented as per Criteria 1 and 4, above.
11. *Appropriate secure access to identifying data.* Where necessary, the data custodian must be able to: (i) access identifying data, and; (ii) link those data back to source data, derived data and biosamples. This linkage facility is critical for error correction, in case of withdrawal of consent, for feedback of clinically relevant findings and in managing some longitudinal data.
12. *Appropriate protection of identifying data.* Although individually identifying data must sometimes be accessible (see 11) they should not be released unless a suitable body deems it absolutely necessary (e.g. for use in a clinical setting or because an important scientific question demands it). Such release may result in the identification of individual study participants and/or information about them being released into the public domain.

### 3.4 Data Safe Haven Criteria are context specific

Crucially, the particular actions required to ensure that each of these criteria is met are *context-specific.* For example, in relation to Criterion 8—which focuses on the appropriate management of data that are potentially identifying - even data items that are individually non-disclosive can become highly identifying if combined together (Golle, 2006). At the same time, it is sometimes *necessary* for scientific reasons to release data that have a relatively high risk of disclosure, e.g. the use of anonymization and de-identification mechanisms can sometimes vitiate the utility of data for certain important research purposes (Erlich and Narayanan, 2014). Taken together, these observations imply: (i) a *zero* risk of disclosure is an unattainable objective and should never be promised, and; (ii) it may well be reasonable to try to strike a balance between the scientific value of a particular set of data, and the risk that it may lead to disclosure. Where that balance should lie might be expected to vary with the importance of the scientific question being addressed and the potential impact of an individual being identified and his/her data being revealed. For example, it may well differ between data generated from a study of HIV/AIDS, where individual disclosure may be highly stigmatizing, and data from a cohort study focusing on childhood asthma. Crucially, determination of such questions of the balance of social and scientific good cannot be left to one set of stakeholders, be they the data custodian, user, participant or other stakeholder. All perspectives are relevant.

### 3.5 A Data Safe Haven does not operate in isolation

Although we believe that in order for an entity to be designated a Data Safe Haven it should satisfy all criteria recommended, the entity itself need not wholly be responsible for all the work underpinning each criterion. Thus, the European Genome/Phenome Archive and the UK Data Archive would both be viewed as Data Safe Havens, and both hold data generated by the 1958 Birth Cohort. But, for all but straightforward access requests, the oversight of access to biomedical data from 1958BC is actually enacted elsewhere—through the Access Committee for CLS Cohorts. A Data Safe Haven must address all criteria in a manner consistent with the nature and purpose of the data it holds—but some criteria may reasonably be met via systems and mechanisms that are managed elsewhere.

### 3.6 Other characteristics that supplement Data Safe Haven criteria

Other characteristics might also be considered desirable for a repository to exhibit. These include: (i) *timeliness*—ensuring that applications are turned round with sufficient speed to avoid inconveniencing applicants; and (ii) *simplicity*—avoiding an application mechanism that is so complex that it puts potential applicants off from applying.

## 4 Conclusion: so what *is* a Data Safe Haven?

This paper claims that, regardless of how the concept of a Data Safe Haven may originally have emerged (Directorate of Information Services MEL(1992)42, 1992; NHS Research Capability Programme, 2008) and regardless of its potential legal use in certain jurisdictions (Caldicott, 2013), the term has evolved in meaning over time and as it has started to be employed world-wide. Its common (intuitive) usage is now consistent with a broad definition that, in effect, states that a Data Safe Haven is a data repository in which biomedical and/or social data can be stored and accessed in a manner that reliably maintains their fidelity and quality but also ensures that the data are ‘safe’ in the sense that all relevant social expectations and ethical and legal controls on their use and dissemination are appropriately met. Crucially, it is important to note that the entities that were first called Data Safe Havens by the UK NHS in the 1990s undoubtedly meet this definition, but they are now just one class of Data Safe Haven amongst a rich array of alternatives.

As things currently stand, with no formal definition of the term Data Safe Haven, any repository could arguably decide to award itself the title. But the term is so useful—and potentially so potent as a tool in engaging the wider community and enhancing understanding of health-related data management—that such loose treatment would weaken its value. To provide greater clarity of the dimensions of a Data Safe Haven, we have described a series of 12 criteria that we believe should be met before a repository can claim the status of a Data Safe Haven. We are sure that this particular set of criteria could be improved upon—and perhaps should be discussed and agreed upon at an international forum, possibly convened by the Global Alliance for Genomics and Health. Yet, we are convinced that not only is an agreed set of such criteria necessary, but also that it is achievable. Crucially, whether a particular repository should or should not be viewed as a Data Safe Haven is strictly dependent on the classes of data it might hold. For example, ethical, legal and quality control criteria, and the spectrum of *bona fide* data users, may vary markedly, and entirely appropriately, between: (i) complex phenotypic data collected by a research-focused cohort study; (ii) routine hospitalization data generated by a health service; (iii) genotypic data arising from a large population-based set of healthy controls, and; (iv) linked data integrating primary care records with educational achievement; and (v) data of potential commercial sensitivity that big Pharma may want to leverage in a pre-competitive space between companies.

In our view, the appropriate definition of a Data Safe Haven which is consistent with the ways in which the term is widely used depends primarily on a repository ensuring that its systems, mechanisms, and policies are transparent, comprehensive and rigorous when judged against the appropriate criteria of data quality and safety that apply to the particular data that it holds, and are appropriately audited to ensure ongoing consistency with those same criteria. If such a definition is to be meaningful, there is a need to develop national and international programs and evaluation mechanisms to enable a formal status of Data Safe Haven to be awarded.

## References

[btv279-B1] Academy of Medical Sciences. (2013) Realising the potential of stratified medicine. Academy of Medical Sciences, London.

[btv279-B2] Academy of Medical Sciences. (2014) Data in Safe Havens. Academy of Medical Sciences, London.

[btv279-B3] Adams S. (2013) GPs boycott big brother data plan. Mail on Sunday.

[btv279-B4] Administrative Data Taskforce. (2012) The UK Administrative Data Research Network: Improving Access for Research and Policy.

[btv279-B5] Anderson R. (1996) Security in Clinical Information Systems.

[btv279-B6] Beauchamp T. Childress J. (2001) Principles of biomedical ethics Oxford University Press, New York.

[btv279-B7] Calabresi G. Bobbitt P. (1978) Tragic Choices. Norton, New York.

[btv279-B8] Caldicott F. (2013) Information: To share or not to share? The Information Governance Review Department of Health, Crown copyright.

[btv279-B9] Care Record Development Board. (2007) Report of the Care Record Development Board Working Group on the Secondary Uses of Patient Information.

[btv279-B10] Chi K.R. (2013) Data Drive. In: LABX Media Group, Midland, ON: The Scientist Magazine.

[btv279-B11] Collins R. Biobank Steering Committee UK (2007) UK Biobank: Protocol for a Large-Scale Prospective Epidemiological Resource. UK Biobank Coordinating Centre, Manchester.

[btv279-B12] Davies J. (2002) Towards the Semantic Web: Ontology-Driven Knowledge Management. John Wiley & Sons, Inc. Chichester, West Sussex.

[btv279-B13] Di Iorio C.T.*.* (2013) Cross-border flow of health information: is 'privacy by design' enough? Privacy performance assessment in EUBIROD. Eur. J. Public Health*,* 23, 247–253. 22562711 10.1093/eurpub/cks043

[btv279-B14] Dilger A. (2013) System and method for end-to-end data integrity in a network file system. Patents, U.S., ed. United States: Oracle International Coorporation, p. 20.

[btv279-B15] Directorate of Information Services MEL(1992)42. (1992) Guidance for the Handling of Confidential Personal Health Information in the Contracting Environment.

[btv279-B16] Doll R. Peto R. (2001) Rights involve responsibilities for patients. BMJ (Clinical Research Ed) 322, 730. PMC1119909 11264225

[btv279-B17] Dwork C. (2006) Differential Privacy. In: Bugliesi M. (eds) 33rd International Colloquium: Automata, Languages and Programming 2006. Part 2 pp. 1–12 Springer, Heidelberg.

[btv279-B18] Erlich Y. Narayanan A. (2014) Routes for breaching and protecting genetic privacy. Nat. Rev. Genet.*,* 15, 409–421. 24805122 10.1038/nrg3723 PMC4151119

[btv279-B20] Gaye A.*.* (2014) DataSHIELD: taking the analysis to the data, not the data to the analysis. Int. J. Epidemiol.*,* 43, 1929–1944. 25261970 10.1093/ije/dyu188 PMC4276062

[btv279-B21] Geels F.W. (2005) Technological Transitions and System Innovations: a Co-evolutionary and Socio-technical Analysis. Edward Elgar Publishing, Cheltenham, Gloucestershire.

[btv279-B22] Global Alliance for Genomics and Health. (2014) Framework for Responsible Sharing of Genomic and Health-Related Data. McGill University, Montreal.

[btv279-B23] Golle P. (2006) Revisiting the uniqueness of simple demographics in the US population. In: Proceedings of the 5th ACM Workshop on Privacy in Electronic Society. ACM, p. 77–80.

[btv279-B24] Hansson M.G. (2010) Need for a wider view of autonomy in epidemiological research. BMJ (Clinical Research Ed)*,* 340, c2335. 10.1136/bmj.c2335 20444822

[btv279-B25] Karr A.*.* (2007) Secure, privacy-preserving analysis of distributed databases. Technometrics*,* 49, 335–345.

[btv279-B26] Karr A. Reiter J. (2014) Using statistics to protect privacy. In: Lane J. (eds.) Privacy, Big Data, and the Public Good Frameworks for Engagement. Cambridge University Press, Cambridge.

[btv279-B27] Kelsey T. (2013) Transparency in the NHS not only saves lives, it is a basic human right. The Guardian 12th March 2013.

[btv279-B28] Lecouturier J.*.* (2008) Clinical research without consent in adults in the emergency setting: a review of patient and public views. BMC Med. Ethics*,* 9, 9. 18445261 10.1186/1472-6939-9-9 PMC2390563

[btv279-B29] Lohr S. (2012) The age of big data. New York Times*.* p. 11.

[btv279-B30] Murtagh M.J. Hepworth J. (2003) Feminist ethics and menopause: autonomy and decision-making in primary medical care. Soc. Sci. Med.*,* 56, 1643–1652. 12639581 10.1016/s0277-9536(02)00172-7

[btv279-B31] NHS Connecting for Health. (2009) Pseudonymisation Implementation Project (PIP). Reference Paper 2. Guidance on Business Processes and New Safe Havens. London.

[btv279-B32] NHS Research Capability Programme. (2008) Case for ‘Honest Broker’ and ‘Safe Haven’ Services to Support Research.

[btv279-B33] OECD Expert Group for International Collaboration on Microdata Access. (2014) Final Report.

[btv279-B34] Owen R. (2013) Responsible innovation: managing the responsible emergence of science and innovation in society. John Wiley & Sons, Chichester, West Sussex.

[btv279-B35] Power C. Elliott J. (2006) Cohort profile: 1958 British birth cohort (National Child Development Study). Int. J. Epidemiol.*,* 35, 34–41. 16155052 10.1093/ije/dyi183

[btv279-B36] Shaw J. (2014) Why “Big Data” is a big deal. Harvard Magazine, March 2014

[btv279-B37] Sweeney L. (2002) k-anonymity: A model for protecting privacy. Int. J. Uncertainty Fuzziness Knowl. Syst.*,* 10, 557–570.

[btv279-B38] Taylor M. (2014) Information governance as a force for good? Lessons to be learnt from care.data. ScriptEd*,* 11, 1–8.

[btv279-B39] Thomas R. (2008) Data Sharing Review. ISBN 978-1-84099-204-5. Ministry of Justice, London.

[btv279-B42] Wolfson M.*.* (2010) DataSHIELD: resolving a conflict in contemporary bioscience—performing a pooled analysis of individual-level data without sharing the data. Int. J. Epidemiol.*,* 39, 1372–1382. 20630989 10.1093/ije/dyq111 PMC2972441

[btv279-B43] Wyke A. (2009) Fixing Healthcare: The Professionals' Perspective. Economist Intelligence Unit, Limited, London.

